# Increased incidence of myxedema coma during the COVID-19 pandemic and in the post pandemic era: a single-center case series

**DOI:** 10.1007/s11739-024-03690-9

**Published:** 2024-07-05

**Authors:** Grzegorz Sokołowski, Katica Bajuk Studen, Marta Opalinska, Karolina Wegrzyn, Marcin Motyka, Aleksandra Gilis-Januszewska, Alicja Hubalewska-Dydejczyk

**Affiliations:** 1 Chair and Department of Endocrinology, Jagiellonian University Medical College, ul. Jakubowskiego 2, 30-688, Kraków, Poland; 2 Faculty of Medicine, Department of Internal Medicine, University of Ljubljana, Ljubljana, Slovenia; 3 Department of Nuclear Medicine, University Medical Centre, Ljubljana, Slovenia; 4 Department of Endocrinology, Oncological Endocrinology, Nuclear Medicine and Internal Medicine, University Hospital, Kraków, Poland

**Keywords:** COVID-19, Hypothyroidisms, Myxedema coma

## Abstract

The COVID-19 pandemic was a major challenge for all health care employees, but it was also difficult for patients to gain access to health care services. Myxedema coma (MC) is an extremely rare but potentially fatal endocrine emergency. The aim of the study was to report an increased incidence of life-threatening myxedema coma that occurred in relation to the COVID-19 pandemic. In this paper, we report a cohort of 11 patients with MC who were treated at the University Hospital in Krakow, Poland, in the period from 2015 to 2023. Only 1 case of MC was recorded in the period from 2015 to 2019, and, in the same area, 10 cases of MC were recorded after the start of COVID-19 pandemic until present. Hypothyroidism was diagnosed de novo in 2 (18%) patients; the remaining patients were severely hypothyroid due to therapy non-compliance. Nine patients had primary hypothyroidism, and 2 had central hypothyroidism. Besides longstanding hypothyroidism, an additional precipitating factor for MC was identified in 4 (36%) of the patients. Due to the inaccessibility of parenteral levothyroxine, patients were treated with oral, mostly liquid, form of levothyroxine. The mortality rate in this cohort was 27.2%. In conclusion, the increase of the incidence of MC, which is a life-threatening complication of inadequately treated hypothyroidism, during the COVID-19 pandemic, when resources were limited, and in the post-pandemic era, underlines the importance of adequate communication with patients and of long-term availability of primary care for patients with thyroid disease.

## Introduction

Hypothyroidism is a pathological condition caused by a deficiency of circulating thyroid hormone concentrations [1]. Clinical manifestations of hypothyroidism can range from no signs and symptoms through a wide range of severity to myxedema coma (MC) [1]. MC is an extremely rare but potentially fatal endocrine emergency that requires prompt recognition and treatment [1, 2]. It represents a state of a decompensated hypothyroidism that usually occurs after a period of longstanding, unrecognized, or poorly controlled thyroid hypofunction and is often precipitated by a superimposed systemic illness or precipitating factor [3]. Such factors include infection, trauma, certain medications, hypothermia, congestive heart failure, cerebrovascular incident, metabolic and electrolyte disturbances [3]. Given the rarity of MC, randomized clinical trials are not feasible and recommendations regarding the treatment are based on expert opinion, case reports and small series of patients [2]. Prognosis has been markedly improved with adequate, often initially aggressive, hormone replacement therapy and intensive care support measures, with in-hospital mortality reaching 30–40% in recent series [4–6]. A diagnostic scoring system to differentiate semi-quantitatively between uncomplicated hypothyroidism and severe hypothyroidism (SH) including MC has been proposed [3]. Coronavirus disease 2019 (COVID-19) was characterized as a pandemic in March 2020 by the World Health Organization [7]. Although thyroid cells express angiotensin-converting enzyme 2 (ACE-2), the main protein that functions as a receptor to which SARS-CoV-2 binds to enter host cells, studies have shown that patients with controlled hypothyroidism and hyperthyroidism do not have a higher prevalence of COVID-19 nor do they have a worse prognosis when infected with the virus [8–11]. Nevertheless, several case reports of severe hypothyroidism and MC in patients with COVID-19 have been published [12–14]. Moreover, the COVID-19 pandemic affected not only the health of general population but also the functioning of healthcare systems, imposing significant changes in existing clinical practices. Limited availability of primary care facilities and insufficient communication with patients led to a reduced number of physical and laboratory examinations [15]. This could also lead to missed diagnoses of hypothyroidism and to disturbances in providing the timely supply of the patients’ chronic medication.

In view of this, the following analysis of the recorded cases of MC in a single center in the period before and during/post COVID-19 pandemic was performed.

## Materials and methods

The study was designed as a retrospective analysis. Patients who met the inclusion criteria, i.e. a diagnosis of MC according to the criteria proposed by Popoveniuc et al. [3] (Table 1), were included in the analysis. Patients were identified by searching electronic records of all patients treated at the University Hospital in Krakow, Poland, in the period from 01.01.2015 to 31.01.2024. Patient identification was followed by data collection including clinical and outcome data. Study variables included initial assessment of thyroid function, evaluation of possible MC triggers, disease course, clinical treatment and follow-up.

**Table 1:** Diagnostic scoring system for myxedema coma (3)

Thermoregulatory dysfunction (temperature, C)		Cardiovascular dysfunction	
> 35	0	Bradycardia	
32–35	10	Absent	0
< 32	20	50–59	10
Central nervous system effects		40–49	20
Absent	0	< 40	30
Somnolent/lethargic	10	Other EKG changes	10
Obtunded	15	Pericardial/pleural effusions	10
Stupor	20	Pulmonary edema	15
Coma/seizures	30	Cardiomegaly	15
Gastrointestinal findings		Hypotension	20
Anorexia/abdominal pain/constipation	5	Metabolic disturbances	
Decreased intestinal motility	15	Hyponatremia	10
Paralytic ileus	20	Hypoglycemia	10
Precipitating event		Hypoxemia	10
Absent	0	Hypercarbia	10
Present	10	Decrease in GFR	10

*EKG* electrocardiogram, *GFR* glomerular filtration rate

a A score of 60 or higher is highly suggestive/diagnostic of myxedema coma; a score of 25 to 59 is suggestive of risk for myxedema coma, and a score below 25 is unlikely to indicate myxedema coma

b Other EKG changes: QT prolongation, or low voltage complexes, or bundle branch blocks, or nonspecific ST–T changes, or heart blocks

## Results

The average number of hospitalisations during the pre-pandemic (2015–2019) and pandemic (2019–2023) periods was 114,710 and 87,400 per year, respectively. Prior to the pandemic period, patients remained under routine outpatient follow-up.

### Case presentation

Eleven patients with a composite diagnosis score of MC of 60 points or more, according to inclusion criteria, were identified.

Of those, all except one patient were admitted to the hospital during or shortly after the COVID-19 pandemic (Table 2).

**Table 2:** The characteristics of hypothyroidism and treatment of patients with myxedema coma

Patient no	Date of admission	History of hypothyroidism	TSH [mIU/L] fT4 [pmol/L] fT3 [pmol/L]	Treatment	Levothyroxine route of administration and maximal dose	Duration of hospitalization [days]
1	Jan 2024	Hashimoto’s thyroiditis; therapy non-compliance	85 2.6 1.82	Levothyroxine	Oral 125 µg	8
2	Dec 2023	Hypothyroidism after thyroidectomy due to goiter (2003); therapy non-compliance	124 0.5 0.6	Levothyroxine, hydrocortisone	Through nasogastral tube 500 µg	Died on day 38 of hospitalization (Staph. sepsis)
3	Nov 2023	None	80.7 0.5 0.6	Levothyroxine, hydrocortisone	Oral 300 µg	Died on day 4 of hospitalization (Heart failure)
4	May 2023	Hashimoto's thyroiditis; therapy non-compliance	171 0.5 0.6	Levothyroxine, hydrocortisone	Oral 300 µg	6
5	Feb 2023	Hypothyroidism of unknown etiology; therapy non-compliance	134 0.5 0.6	Levothyroxine, hydrocortisone	Through nasogastral tube/per rectum 1500µg (1000 ug per rectum + 500 ug through nasogastral tube)	27
6	Jan 2023	Hashimoto's thyroiditis; therapy non-compliance	110 2.0 0.6	Levothyroxine	Oral 100 µg	8
7	Apr 2022	Hashimoto's thyroiditis; therapy non-compliance	17.8 2.6 1.2	Levothyroxine, hydrocortisone	Through nasogastral tube 200 µg	24
8	Dec 2021	Panhypopituitarism; therapy non-compliance,	0.04 10.0 0.9	Levothyroxine, hydrocortisone, dexamethasone	Through nasogastral tube/oral 137 µg	57
9	May 2021	Panhypopituitarism; therapy non-compliance	4.11 4.9 1.7	Levothyroxine, Hydrocortisone	Oral 75 µg	17
10	Mar 2021	Hypothyroidism of unknown etiology; therapy non-compliance	57 5.4 n/a	Levothyroxine, hydrocortisone	Through nasogastral tube 150 µg	102
11	Dec 2019	None	36 0.9 0.7	Levothyroxine, Hydrocortisone	Through nasogastral tube 400 µg	Died on day 10 of hospitalization (Cardiac arrest)

Normal range: TSH: 0.270–4.200 uIU/mL, FT3: 3.1–6.8 pmol/L, FT4: 12.0–22.0 pmol/L, n/a–non-available

The characteristics of hypothyroidism and the dose and the route of administration of levothyroxine are depicted in Table 2. The clinical characteristics of the patients at admission and myxedema coma (MC) score for the patients are reported in Table 3.

**Table 3:** Clinical characteristics at admission and myxedema coma (MC) score* for patients with myxedema coma

No	Sex	Age [years]	Central nervous system impairment	Precipitating event	eGFR [mL/min]	Na [mmol/L]	Glucose [mmol/L]	Temp [°C]	Heart rate [/min] Hypotension [< 90/60 mm Hg] Pericardial effusion	ECG change	Chest X-ray findings	Gastro-intestinal findings	Hypercarbia	Hypoxemia	MC score*
1	F	71	Obtundation	Head injury	57	144	7.2	36.6	56 − −	−	Cardiomegaly Pleural effusion	Pain; paralytic obstruction	+	−	75
2	F	79	Coma	Sepsis	57	143	5.9	34.8	60 + −	−	−	−	−	+	90
3	M	45	Obtundation	Diarrhea	67	116	1.9	36.5	82 + +	Low voltage	Cardiomegaly pleural effusion and edema	Diarrhea, jaundice	−	+	135
4	F	64	No	Urinary tract infection	36	139	7.4	36.4	40 + +	Low voltage	Cardiomegaly	–	+	−	85
5	F	85	Obtundation	SARS-CoV-2 infection	44	141	4.2	34.5	52 − −	−	Pleural effusion	Pain, constipation, paralytic ileus	+	−	100
6	F	66	Obtundation	Transient ischemic attack	42	136	7.7	36.6	60 − +	−	Cardiomegaly	Pain, constipation	−	−	75
7	M	68	Stupor	Infection (Otitis media)	57	132	5.39	33.2	50 + −	Non specific ST-changes	Cardiomegaly pleural effusion	Constipation	+	+	140
8	F	60	Coma	SARS-CoV-2 infection	> 90	116	3.9	36.1	56 + −	−	−	−	−	+	90
9	M	46	Coma	„Detox diet”, diarrhea	33	142	6.87	36.6	65 − −	−	Pleural effusion	Pain, diarrhea	−	−	65
10	F	66	Somnolence	SARS-CoV-2 infection	72	141	4.75	36.0	70 − −	−	Cardiomegaly pleural effusion	−	−	+	95
11	F	74	Coma	Infection of unknown origin	74	144	6.7	30.0	50 + +	−	Cardiomegaly	−	+	+	145

*Myxedema coma score according to Popoveniuc et al. [3]. A score of 60 or higher is highly suggestive/diagnostic of myxedema coma; a score of 25 to 59 is suggestive of risk for myxedema coma, a score below 25 is unlikely to indicate myxedema coma. Normal range: Na 135–145 mmol/L, Glucose 3.30–5.60 mmol/L, eGFR > 90 mL/min/1.73m2, [ +]—symptom present, [−]—symptom absent

Hypothyroidism was diagnosed de novo in 2 (18%) patients; the remaining were severely hypothyroid due to therapy non-compliance. Nine patients had primary hypothyroidism, and 2 had central hypothyroidism. In patient 7, the concentration of the measured TSH was lower than expected regarding the concentrations of free T4 and free T3. At the time of admission, further diagnostics of possible hypopituitarism due to simultaneous treatment with stress doses of glucocorticoids was not possible.

Due to the inaccessibility of parenteral levothyroxine, patients were treated with liquid levothyroxine *per os*, through nasogastral tube or *per rectum*. In addition to levothyroxine, hydrocortisone in stress doses was prescribed in the majority of the patients. According to the clinical state, patients were also treated with additional intensive care support measures.

The maximum daily dose of levothyroxine used to treat MC was 75 to 1,500 ug. In most patients, oral or nasogastric tube administration was sufficient; in one patient, due to paralytic bowel obstruction, per rectum administration was necessary. The time to normalization of free T4 ranged from 5 to 70 days. The mortality rate in the described cohort was 27.2%.

## Discussion

We report a single-center experience of an increased incidence of MC in the time period from the beginning of COVID-19 pandemic until present. In our cohort of patients, MC occurred due to limited access to primary/specialist care and fear of SARS-CoV-2 infection, leading to missed follow-up appointments or undiagnosed hypothyroidism; some patients were hospitalised for serious COVID-19-related or non-COVID-19-related conditions overlapping with hypothyroidism. Although several case reports of severe hypothyroidism and MC diagnosed in patients with COVID-19 have been published [12–14], we have identified only one case report of an exacerbation of hypothyroidism leading to MC during the COVID-19 lockdown [16]. To our best knowledge, this is the first report of an increased incidence of MC in the period of COVID-19 pandemic, when access to primary care services was very limited, and in the early post-pandemic era, when long-term consequences of the lock-down have been expected to manifest.

Several large population-based cohort studies have evaluated the outcomes of acute SARS-CoV-2 infection among individuals with pre-existing treated hypothyroidism and suggested that these patients do not have increased susceptibility to SARS-CoV-2 infection nor increased risk of adverse COVID-19 related outcomes compared with patients with COVID-19 and normal thyroid function [8–11, 17–20]. Moreover, thyroid dysfunction associated with COVID-19 has been recognized, secondary to direct and indirect effects on the hypothalamic-pituitary-thyroid axis [21]. Typically, three patterns of abnormal thyroid function can be identified: non-thyroid illness syndrome (the most consistently reported pattern), patterns suggestive of thyroiditis, and pre-existing autoimmune thyroid disorders. Regarding autoimmune thyroid disorders, there is no firm evidence of overrepresentation of Hashimoto's thyroiditis in cohorts of patients with COVID-19 compared with general population [21]. Therefore, the increased incidence of MC in our cohort is most probably associated with limited access to primary health care services and inadequate patient information and not direct SARS-CoV-2 infection.

Besides thyroid hormone concentration measurement, the objective clinical severity of hypothyroidism of the patients included in our cohort was assessed by the MC score, as proposed by Popoveniuc et al. (Table 1) [3]. In addition to the state of consciousness, the parameters composing this score include the presence of a precipitating factor, body temperature, electrolyte, metabolic, cardiovascular, respiratory and gastrointestinal disturbances. Moreover, it has been suggested that the term MC can be misleading, since not all patients with severe hypothyroidism present with coma. Hemodynamic and ventilation failures are also frequent clinical presentations associated with worse outcomes [4, 5, 22, 23]. The validity of the MC score for diagnosing severe hypothyroidism was confirmed by two recent larger studies for patients with primary but also central hypothyroidism [4, 23].

Hypothyroidism was diagnosed de novo in two patients; in the majority, the severe form of hypothyroidism was due to a long-standing therapy non-compliance. Since the patients were severely hypothyroid, we were unable to establish the duration of non-compliance from their clinical history with precision. Discontinuation of levothyroxine was also described as one of the most common triggers for critically ill severe hypothyroidism in a large French cohort [4].

In MC, both the initial dose and the route of administration of levothyroxine are still a matter of debate as aggressive levothyroxine replacement at the onset of SH treatment may increase the risk of myocardial infarction or arrhythmias [24]. Therefore, levothyroxine replacement in the context of SH should be pragmatic and adapted to the patient’s age, medical history and general condition. Due to the inaccessibility of parenteral levothyroxine, our patients were treated with oral, mostly liquid, levothyroxine, as previously described [23, 25, 26, 29] and with the use of a liquid form of levothyroxine in rectal infusions, which may provide additional clinical benefit in patients with malabsorption secondary to paralytic intestinal obstruction. The lack of clinical improvement associated with the low initial levothyroxine dose, used in some patients before admission to the endocrinology department, confirms the need for initial treatment with high levothyroxine doses, considering the possibility of impaired intestinal absorption. The mortality of our cohort was similar as recently reported [4, 5].

A long time lag between the start of a health crisis such as the COVID-19 pandemic and the manifestation of severe clinical consequences of inadequate management of hypothyroidism was expected since most effects of thyroid hormones are mediated through binding to nuclear receptors and modulating the expression of thyroid hormone-responsive genes [27]. Therefore, usually there is a long period between the onset of hypothyroidism and overt clinical picture. In addition, the initial symptoms and signs of hypothyroidism can be vague, wide-ranging and non-specific [27]. MC is defined as an extremely rare form of severe hypothyroidism that usually occurs after a long period of unrecognized or poorly controlled thyroid hypofunction [3].

The guidance on the management of thyroid dysfunction during the COVID-19 pandemic, published with the aim to optimize patient care during the COVID-19 health crisis, suggested remodelling some of the endocrine services to telephone and video consultations and remote monitoring services; however, it did foresee that patients with thyroid dysfunction will not be managed as closely as in a non-crisis situation [7]. The Polish National Health Program for 2021–2025 emphasized that the COVID-19 epidemic has caused a negative synergistic effect [15]. The Patients Rights Ombudsman’s Report has shown that exclusion of some general hospitals and their transformation into COVID-19-dedicated hospitals has made it much more difficult for patients using these units to access health services and has heightened their concerns about the continuity of treatment [28]. In addition, the spread of the SARS-CoV-2 virus has affected the use of medical services in Poland. Comparing data from July 2018 and July 2020, a Center for Public Opinion Research found that there was a clear increase in the overall number of people who did not receive any treatment and examinations (from 12 to 30%) [29]. In a study aimed to assess patients’ perspectives on the impact of the COVID-19 pandemic on the treatment and diagnostic process in Poland, 64.3% of the respondents rated the process negatively [15]. Increased focus on the medical problems associated with COVID-19 and the gradual development of symptoms, inadequate information and the lack of sufficient communication with patients and their relatives have been identified as the main impediments.

One of the limitations of our study is the scarcity of included cases, limiting its utility. To expand the case pool to be able to generate more robust evidence, a multi-centre study with pooled data or a study based on the national registry data would be feasible. Furthermore, given the retrospective nature of our study, the possibility of underreporting of MC cases before the COVID-19 pandemic could represent a possible bias. However, the same search criteria in the hospital electronic registry for both the pre-pandemic period and the pandemic/post-pandemic period were employed.

## Conclusion

We report an increased incidence of MC, an extremely rare but life threatening complication in patients with hypothyroidism, in a single center in the period during and after the COVID-19 pandemic. Since there is no firm evidence of overrepresentation of hypothyroidism in patients with COVID-19 compared with general population, this increased incidence is most probably associated with limited access to primary care services and inadequate patient information. Our findings indicate the necessity of providing an adequate care for patients with thyroid disease aiming to reduce deaths from a common, relatively easy-to-treat disease.

## Summary

The COVID-19 pandemic posed a huge challenge for all health care workers, but patients also had difficult access to health care. Fear of COVID-19 infection was also an important factor affecting patients’ compliance. Before the COVID-19 pandemic era, myxedema coma was an extremely rare clinical condition. We present the cases of 10 patients who developed life-threatening hypometabolic coma during the COVID-19 pandemic, when access to primary care was very limited, and in the early post-pandemic era, when long-term consequences of the lock-down are expected to manifest.

We present the cases of 10 patients who developed life-threatening hypometabolic coma during COVID-19 pandemic. It developed due to slowly developing symptoms of hypothyroidism leading to underdiagnosis, discontinuation of replacement therapy, or due to overlapping life-threatening SARS-Cov-2 infection symptoms. Our findings highlight the need to develop appropriate care for patients with thyroid disease to reduce deaths from a common and relatively easy to treat condition.
